# Predictive Factors for Pediatric Craniopharyngioma Recurrence: An Extensive Narrative Review

**DOI:** 10.3390/diagnostics13091588

**Published:** 2023-04-28

**Authors:** Anastasios Serbis, Vasiliki Rengina Tsinopoulou, Anastasia Papadopoulou, Savvas Kolanis, Eleni I. Sakellari, Kosmas Margaritis, Eleni Litou, Stergianna Ntouma, Styliani Giza, Eleni P. Kotanidou, Assimina Galli-Tsinopoulou

**Affiliations:** 1 Department of Pediatrics, School of Medicine, University of Ioannina, St. Niarhcos Avenue, 45500 Ioannina, Greece; 2 Unit of Pediatric Endocrinology and Metabolism, 2nd Department of Pediatrics, School of Medicine, Faculty of Health Sciences, Aristotle University of Thessaloniki, AHEPA University Hospital, Stilponos Kyriakidi 1, 54636 Thessaloniki, Greece

**Keywords:** craniopharyngioma, recurrence, risk factors, children, adolescents, youth

## Abstract

Despite being classified as benign tumors, craniopharyngiomas (CPs) are associated with significant morbidity and mortality due to their location, growth pattern, and tendency to recur. Two types can be identified depending on age distribution, morphology, and growth pattern, adamantinomatous and papillary. The adamantinomatous CP is one of the most frequently encountered central nervous system tumors in childhood. Our aim was to review the relevant literature to identify clinical, morphological, and immunohistochemical prognostic factors that have been implicated in childhood-onset CP recurrence. Lack of radical surgical removal of the primary tumor by an experienced neurosurgical team and radiotherapy after a subtotal excision has been proven to significantly increase the recurrence rate of CP. Other risk factors that have been consistently recognized in the literature include younger age at diagnosis (especially <5 years), larger tumor size at presentation, cystic appearance, difficult tumor location, and tight adherence to surrounding structures, as well as the histological presence of whorl-like arrays. In addition, several other risk factors have been studied, albeit with conflicting results, especially in the pediatric population. Identifying risk factors for CP recurrence is of utmost importance for the successful management of these patients in order to ultimately ensure the best prognosis.

## 1. Introduction

Craniopharyngiomas (CPs) are rare epithelial tumors that arise along the path of the craniopharyngeal duct [1,2]. The World Health Organization (WHO) classifies them as grade I tumors due to their lack of histological signs of malignancy [3], although malignant transformation has been reported in rare cases [4]. Histologically, there are two main CP subtypes, namely, adamantinomatous and papillary, which differ in age distribution, frequency, biology, and clinical outcome [2,5,6,7]. Adamantinomatous CP predominantly affects subjects younger than 14 years of age and is, therefore, considered the “pediatric” type, accounting for 5–10% of central nervous system (CNS) tumors in this age group [5,7].

Despite recent advances in its diagnostic and therapeutic approach, CP is still a tumor that is difficult to treat, with significant neurological, endocrinological, and visual complications [8], which can lead to poor quality of life for patients and their families [9], and with an excess mortality rate compared to the general population [10,11]. Both morbidity and mortality of CPs are related not only to the primary tumor itself but also to its tendency to recur, even after radical surgical excision [12], with an average time of 3 years from treatment to recurrence [13,14,15].

The quest for reliable markers that could identify those CP patients at increased recurrence risk has been extensive over the past 40 years [16,17]. For example, it is well established that the radical surgical removal of the primary tumor by an experienced neurosurgical team and the use of radiotherapy (RT) significantly reduce the recurrence rate of CP [1,13,18,19,20,21,22]. Other risk factors that have been consistently recognized in the literature include younger age [23,24,25], larger tumor size, difficult location, and tight adherence to surrounding structures [26,27,28,28,29], as well as the histological presence of whorl-like arrays [30,31]. In addition, clinical risk factors, albeit with conflicting results, include the presence of hydrocephalus or other signs of increased intracranial pressure (ICP) [1,16,32], visual disturbances [30,32], and hypothalamic [24,33] involvement. Similarly, several studies of CP molecular features had conflicting results, such as Ki-67, p53 gene, vascular endothelial growth factor [VEGF], and cathepsins [34,35,35,36,37,38,39]. Finally, the presence of calcifications in the primary tumor has been linked with an increased recurrence rate [40,41,42], while the treatment of the patient with recombinant human growth hormone [GH] shows little or no risk [43,44,45].

We aimed to perform a narrative review of the literature focusing on risk factors that increase the likelihood of recurrence of childhood CP published since the early 1980s. Only two relevant systematic reviews have been conducted in adults, which concluded that there is a need for further studies on CP recurrence markers [16,17].

## 2. Materials and Methods

In the present study, conducting a narrative review was chosen on the grounds that, even if a systematic review is of paramount importance in gathering and critically synthesizing all relevant literature on a given topic, narrative reviews are complementary to the research process since they constitute a comprehensive and objective analysis of the current knowledge on a topic, such as CP recurrence.

The literature search on PubMed/Medline database was conducted referring to manuscripts/studies published between 1 January 1980, and 31 January 2023, to identify relevant papers using the following keywords: “craniopharyngioma”, “risk factors”, “regrowth”, “recurrence”, “pediatric”, “child”, “adolescent”. Exclusion criteria were the following: non-English papers; studies comprised exclusively adult study populations or studies in which the age of participants was not clearly defined; editorials and letters to the editor; studies that examined risk factors for other CNS cancer types, and studies of poor quality, e.g., with inappropriate statistical methods, or inadequate patient or treatment data description and follow-up. Clinical case reports, clinical case series, observational studies, and systematic reviews were all included in the initial evaluation. Duplicates and relevance were initially evaluated based on the title and abstract screening. Full-text articles from all relevant studies were retrieved and reviewed. Additional relevant papers that were identified through a manual search of the references from the retrieved articles were also included.

## 3. Results

**Figure 1:**
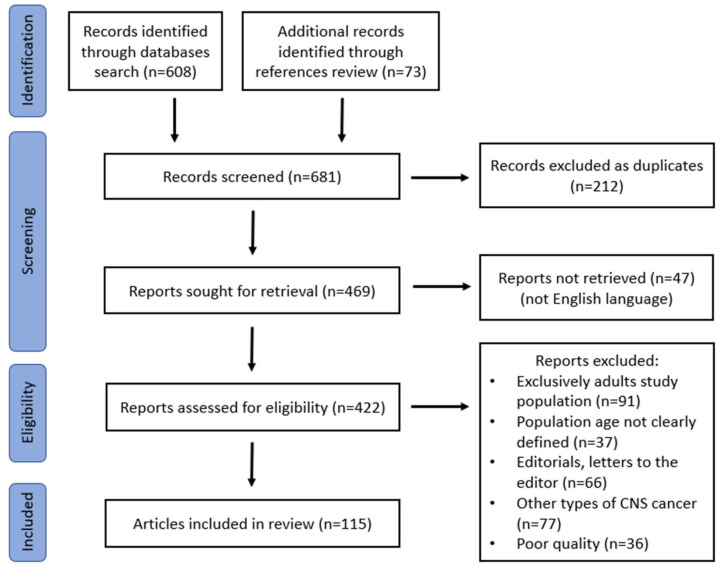
Flowchart of narrative review of the literature (record identification, eligibility, and final inclusion).

The initial literature search identified 608 records, of which 212 were excluded as duplicates. In addition, 47 reports were not available in the English language and were equally excluded. After the manual check of the reference list of the retrieved reports, 73 additional records were deemed relevant (Figure 1). Among the 422 reports that were retrieved and reviewed in total, 307 were excluded for reasons presented in detail in the screening flowchart (Figure 1). In the end, 115 articles were considered pertinent and were included in the current review.

## 4. Discussion

### 4.1. Epidemiological Characteristics and Recurrence

Regarding the patient’s personal history and morphological features of the disease, younger age has been linked in several studies with a higher rate of CP recurrence. For example, in an early large study that included 173 patients (45% children aged <16 years) with CP treated with external RT either alone or following surgery, adjusted for other risk factors for death, and after 12 years of median follow-up, the CP recurrence risk increased in parallel to age of presentation. More specifically, the relative risk was 1.0 for the age group < 16 years, 0.58 for the age group of 16–39, and 0.40 for patients 40 years and older [25]. In a more recent study from France with 171 patients (65 with childhood-onset and 106 adult-onset CP), diagnosis before the age of 10 years was an independent risk factor for recurrence [24]. In addition, it was associated with a higher incidence of obesity, blindness, and panhypopituitarism, and with developmental complications, since among the early onset group, only 40.7% of patients had adequate school performance or professional life compared to 72.4% of patients with later onset of the disease [24].

Similar were the results of a smaller, more recent study by Šteňo et al. [46], which included 38 children and 63 adults with CP treated with RT and/or surgery and were followed for a mean of >10 years. This study showed that the recurrence rate was higher in children compared to adults (39.5% vs. 22.2%, respectively), and this difference persisted even for patients with radical tumor excision (36.7% vs. 14%, respectively). Some studies have identified an even younger age (<5 years) to be an independent risk factor for CP recurrence [23,40,47]. The larger tumor size at presentation and its adhesion to the surrounding structures, as well as a more aggressive behavior of CP in younger patients, could explain the higher rate of CP recurrence in childhood-onset CP. In addition, delayed or nonuse of RT, especially in earlier studies, has been implicated in higher CP recurrence rates in children [16,23,24]. Nevertheless, a few studies have not found an association between CP recurrence risk and younger age [32,48,49].

Male sex has been associated in some studies with an increased risk of CP recurrence. For example, in their large retrospective study, Gautier et al. [24] found that CP recurrence was more common in male subjects. Similarly, Mortini et al. found that the male sex was an independent risk factor for recurrence in a group of adult and pediatric patients [22]. On the contrary, several authors have found no correlation between the male sex and the risk of CP recurrence either in adults or in children [14,29,30,48,49]. Since no plausible pathomechanism has been suggested to explain this male–female difference in recurrence rate and relevant data are inconsistent and scarce, especially in the pediatric population, a strong association between the male sex and increased recurrence risk cannot be established [16].

### 4.2. Morphological Features of the Tumor and Recurrence Rate

Several morphological features of CP, such as size, location, adherence to surrounding tissues, as well as its consistency, have been studied in relation to its recurrence risk (Table 1). Firstly, large tumor size at presentation (>3–5 cm) has been identified as an independent risk factor. As an example, in an early study with 61 children (median age 7.5 years) treated for CP in Boston between 1970 and 1990 and followed up for a median of 10 years, 5 of 6 patients with tumors ≥ 5 cm experienced recurrences while only 6 of 30 recurred when the tumor was <5 cm [19]. Similar were the results of another study by De Vile et al. [23], which showed that large tumor size, young age, and severe hydrocephalus were predictors of tumor recurrence in a cohort of 75 children treated for CP.

Tumor size ≥ 5 cm was also found to be an independent risk factor for CP recurrence in a more recent retrospective analysis of 86 children younger than 21 years of age [15]. In another study by Gupta et al. [26], 116 pediatric craniopharyngiomas (68 boys and 48 girls; age range 1.6–18 years) were reviewed and showed that tumor size > 4 cm was strongly associated with tumor recurrence. Similar were the results of two more recent studies [50,51]. The association of larger tumors with increased recurrence risk seems to be multifactorial. Larger tumors occupy larger intracranial compartments and invade surrounding anatomical structures and are, therefore, more difficult to remove completely [23,28]. For example, in a study of 309 patients with CP from China, in which the tumor size was 2–9 cm in diameter, patients with larger tumors showed a higher recurrence rate due to partial or subtotal resection [41]. Further, larger tumors increase the possibility of even a small tumor remaining after surgical excision, which increases the regrowth–recurrence risk [18,28] and possibly in a relatively short time after the first surgical intervention [15]. In addition, larger tumors present more frequently with severe hydrocephalus, which also precludes a total resection, thus increasing the recurrence risk.

Tumor location is another factor that has been associated with increased CP recurrence risk. Several investigators have observed that certain CP locations are more prone to recur, possibly due to difficulty in total resection due to attachment to and/or infiltration of the hypothalamus, attachment to important vascular structures, or involvement of the third ventricle [18,28,29,52]. Kim et al., for example [14], investigated retrospectively 36 children (age range 1–15 years) that had undergone radical excision without RT for a mean follow-up period of 52 months. They found that tumor location was the single most significant clinical predictor of recurrence since the 5-year recurrence-free survival rate was 39% for those who had an intrasellar tumor component and 81% for those who did not (*p* < 0.05). Anatomical structures adjacent to the tumor, such as the optic chiasm, the hypothalamus, and the pituitary stalk, were the most common sites for adhesion, and residual tumor in the optic apparatus was more likely to relapse [14]. Moreover, intracranial sites with intracellular compartments, especially in the vicinity of the pituitary fossa, were also associated with a high probability of relapse [14]. Several authors consider CP location relative to the hypothalamus so important as to support the need for a hypothalamus-referenced classification of CP [53,54,55].

A third morphological characteristic of CP that has been associated with an increased risk of recurrence is the degree of tumor adherence to surrounding vascular or neural structures. Although difficult to define precisely, tumor adherence refers to the neurosurgeon’s ability to find a clear plan for adequate tumor resection [56]. Three factors define the type of adherence, namely, to which intracranial structures the tumor is attached, its adherence morphology, and its strength [56]. Several studies have shown a relationship between these factors and the success of tumor removal. The strongest and most extensive adhesions in the hypothalamus that preclude any attempt to perform a safe total removal are observed in CPs arising from the suprasellar cistern and secondarily invade the third ventricle, and in those with subpial growth at the third ventricle floor [1,18,28,29,41,55,57,58]. Indeed, in the series of children with CP by Tomita et al. [29], only 33% of CPs associated with the third ventricle were completely removed, in contrast to nearly 70% of extraventricular tumors. Similarly, Fahlbusch et al. [28] reported a much lower rate of total removal of intraventricular CPs compared to the general rate (21% vs. 50%, respectively). Indirect evidence of the role of tumor adherence in CP recurrence comes from the observation that the usual location of CP recurrences is frequently the anatomic areas where the primary tumor presented the tighter adherence [14,41]. The importance of tumor adherence regarding the surgical risk of hypothalamic injury, surgical removal extent, and, thus, the risk of tumor recurrence has led some authors to develop a comprehensive descriptive model based on the location, morphology, and strength of tumor attachment. This model is divided into five hierarchical levels of increasing severity, namely, mild, moderate, serious, severe, and critical, and can be used to anticipate the surgical risk of hypothalamic injury and to plan the degree of removal accordingly [56,59].

Finally, tumor consistency, meaning cystic, solid, or mixed cystic/solid tumor, has been associated with the risk of CP recurrence. A few studies in both adults and children have shown that the removal of cystic CPs is associated with a higher recurrence rate compared to the removal of predominantly solid CPs [16,26,60]. A possible explanation for this is the difficulty in removing an intact cystic tumor capsule during surgical removal.

**Table 1:** Categorized risk factors that have been studied in childhood-onset CP recurrence. Each factor is colored according to the following: dark blue for factors strongly protective against recurrence; light blue for factors that most probably have no association with recurrence; dark red as strongly heightened risk; light red as weakly heightened risk; grey for factors with inconclusive data.

Category	Risk Factor	Association Found	Study
**Epidemiological features**	Younger age	Increases the risk	Rajan et al. [25], De Vile et al. [23] Fisher et al. [40], Gautier et al. [24], Šteňo et al. [46], Drimtzias et al. [47]
		No association	Duff et al. [32], Lena et al. [48], Al Shail et al. [49]
	Male sex	Increases the risk	Gautier et al. [24], Mortini et al. [22]
		No association	Kim et al. [14], Lena et al. [48], Tena-Suck et al. [30], Tomita et al. [29], Al Shail et al. [49]
**Morphological features**	Large size	Increases the risk	Hetelekidis et al. [19], de Vile et al. [23], Elliot et al. [15] Gupta et al. [26], Shi et al. [41], Weiner et al. [27], Yosef et al. [50], Kobayashi et al. [51]
	Tumor location [e.g., third ventricle involvement]	Increases the risk	Kim et al. [14], Fahlbusch et al. [28], Tomita et al. [29], Van Effenterre et al. [18], Kim et al. [14]
	Tumor adherence to surrounding tissues	Increases the risk	Fahlbusch et al. [28], Tomita et al. [29], Karavitaki et al. [1], Pan et al. [57], Pascual et al. [55], Pascual et al. [58], Shi et al. [41], Effenterre et al. [18]
	Cystic tumor consistency	Increases the risk	Gupta et al. [26], Lee et al. [60], Prieto et al. [16]
**Clinical presentation**	Hydrocephalus (increased ICP)	Increases the risk	Prieto et al. [16], DeVile et al. [23], Gautier et al. [24]
		Some association	Kim et al. [14], Tomita et al. [29], Gupta et al. [26], Al Shail et al. [49], Poretti et al. [9], Liubinas et al. [61], Fahlbusch et al. [28]
		No association	Duff et al. [32], Karavitaki et al. [1], Kim et al. [14], Puget et al. [44]
	Visual disturbances at presentation	Increases the risk	Duff et al. [32], Lee et al. [62]
		No association	Shail et al. [49], Tena-Suck et al. [30], Drimtzias et al. [47]
	Hypothalamic involvement	Some association	Vinchon et al. [33], De Vile et al. [23], Poretti et al. [9]
		Decreases the risk	Gautier et al. [24]
	Hormonal-related symptoms	Increases the risk	Tena-suck et al. [30], Rogers et al. [63], Erfurth et al. [64]
		Better outcome	Gautier et al. [24]
**Histological features**	Adamantinomatous vs. papillary CP	Adamantinomatous increases recurrence risk	Adamson et al. [65], Szeifert et al. [66], Crotty et al. [67], Tavangar et al. [68]
		No difference between the two types	Duff et al. [32], Eldevik et al. [69], Gupta et al. [26], Kim et al. [14], Minamida et al. [70], Tena-Suck et al. [30], Weiner et al. [27], Prieto et al. [16], Agozzino et al. [35], Zygourakis et al. [71]
	Presence of finger-like epithelial protrusions	Increases the risk	Adamson et al. [65], Weiner et al. [27]
		No association	Duff et al. [32], Gupta et al. [26], Tena-Suck et al. [30]
	Presence of whorl-like arrays	Increases the risk	Stache et al. [31], Tena-Suck et al. [30],
	Intense reactive peritumoral gliosis	Possible risk increase	Pascual et al. [58], Qi et al. [57], Weiner et al. [27], Bartlett [72]
		Possible positive effect on number of recurrences	Vile et al. [23], Minamida et al. [70], Tomita et al. [29], Weiner et al. [27], Adamson et al. [65], Prieto et al. [16]
**Molecular features**	High Ki-67 expression	Increases the risk	Nishi et al. [73], Rodriguez et al. [34], Prieto et al. [16], Raghavan et al. [74], Izumoto et al. [75], Anegawa et al. [76], Guadagno et al. [77], Xu et al. [78]
		No association	Agozzino et al. [35], Kim et al. [14], Park et al. [79], Losa et al. [80], Duo et al. [81], Raghavan et al. [74], Yalçın et al. [82], Moszczyńska et al. [83]
	p53 gene loss of function	Increases the risk	Tena-Suck et al. [30]
		Possible association	Ishida et al. [36], Lefranc et al. [39], Prieto et al. [16], Ujifuku et al. [84]
		No association	Momota et al. [85], Yalcin et al. [82]
	Vascular endothelial growth factor (VEGF)	Increases the risk	Liu et al. [86], Sun et al. [87], Agozzino et al. [35], Xia et al. [88], Elmaci et al. [37]
		No association	Xu et al. [89]
	Expression of RAR isotypes and cathepsins	RARγ increases the risk	Lubansu et al. [38], Lefranc et al. [39]
	Hormones and their receptors	Possible association	Hofmann et al. [90], Li et al. [91]
		No association	Martínez-Ortega et al. [92]
**Therapeutic approach**	Presence of tumor remnants after excision	Increases the risk	Amendola et al. [93], Baskin et al. [94], Cabezudo et al. [95], Carmel et al. [96], Crotty et al. [67], De Vile et al. [23], Duff et al. [32], Elliot et al. [15], Fahlbusch et al. [28], Eldevik et al. [69], Gautier et al. [24], Gupta et al. [26], Hetelekidis et al. [19], Hoffman et al. [13], Karavitaki et al. [1], Khafaga et al. [97], Lena et al. [48], Mortini et al. [22], Puget et al. [44], Schoenfeld et al. [20], Shi et al. [41], Tena-Suck et al. [30], Thompson et al. [98], Tomita et al. [29], Van Effenterre et al. [18], Weiner et al. [27], Yasargil et al. [99], Zuccaro et al. [100]
	Neurosurgical team expertise	Affects the recurrence rate	Mortini et al. [101], Bao et al. [102], Yosef et al. [50], Zygourakis et al. [71], Prieto et al. [16], Tavangar et al. [68]
	Use of radiotherapy after subtotal surgical removal	Decreases the risk	Baskin et al. [94], Cabezudo et al. [95], Carmel et al. [96], Crotty et al. [67], De Vile et al. [23], Duff et al. [32], Eldevik et al. [69], Fisher et al. [40], Hetelekidis et al. [19], Karavitaki et al. [1], Khafaga et al. [97], Mortini et al. [22], Richmond et al. [103], Schoenfeld et al. [20], Stahnke et al. [104], Thompson et al. [98], Tomita et al. [29], Thomsett et al. [105], Weiss et al. [106], Wen et al. [107], Amendola et al. [93], Enayet et al. [108], Stripp et al. [109]
	Presence of calcifications	Increases the risk	Fahlbusch et al. [28], Fisher et al. [40], Zhang et al. 2008 [110], Cheng et al. [111]
		No association	Elliott et al. [42], Drimtzias et al. [47]
	Use of GH replacement therapy	Increases the risk	Taguchi et al. [112], Niu et al. [113]
		No association	Arslanian et al. [114], Olsson et al. [115], Kanev et al. [116], Moshang [43], Karavitaki et al. [117], Rohrer et al. [118], Boekhoff et al. [119], Boguszewski et al. [120], Puget et al. [44], Kim et al. [14], Elliott et al. [15], Clayton et al. [121], Child et al. [122], Darendeliler et al. [45], Moshang et al. [123], Price et al. [124], Smith et al. [125]
		Decreases the risk	Alotaibi et al. [126]

### 4.3. Clinical Presentation at Initial Diagnosis and Recurrence

Clinical manifestations of CP are suggestive of tumor invasion and damage to adjacent tissues. Typical initial CP manifestations include headache and vomiting in 60–80% of cases, hydrocephalus (all as a result of raised ICP), as well as visual deficits, hypothalamic damage, and hormonal-related manifestations (as a result of local invasion to adjacent structures). Of all clinical manifestations of CP, mostly hydrocephalus and visual symptoms have been associated with an increased risk of tumor recurrence (Table 1). Specifically, in a systematic review, it was reported that hydrocephalus at presentation was the unique symptom associated with tumor recurrence; however, this association was characterized as inconclusive as its role remains controversial [16]. In two studies, hydrocephalus at presentation was significantly associated with higher risk of tumor recurrence in children and mixed-age patients, respectively (DeVile et al.: Mann–Whitney U-test: z = −3.15, *p* < 0.002; Gautier et al.: HR: 2.12 95% CI [1.21–3.71], *p* < 0.01) [23,24]. In four other studies, the role of hydrocephalus and raised ICP as risk factors for recurrence were investigated, and both were associated with tumor recurrence since >30% of patients in the recurrence groups had hydrocephalus and intracranial hypertension at presentation (Gupta et al.: 33%; Al Shail et al.: 71.4%). However, no significant association was noted after statistical analysis (Kim et al.: *p* = 0.1408; Tomita et al.: *p* = 0.41915; Gupta et al.: *p* = 0.32; Al Shail et al.: *p* = 0.122) [14,26,29,49]. Other studies have also reported some association between hydrocephalus and tumor recurrence risk but without a detailed statistical analysis [9,15,28,61], and still others did not prove a consistent relationship [1,14,32,44].

Concerning visual deficits, almost half of the juvenile patients are referred to with such symptoms at the time of presentation, most usually as difficulty seeing at school and blurring of vision due to bitemporal hemianopia from optic chiasm compression [127,128]. Several studies have pointed out a possible association between visual symptoms and the risk of CP recurrence. For example, in the study by Duff et al. [32], pediatric patients with visual symptoms at presentation exhibited a higher rate of tumor recurrence (15.1% at 1 year) compared with those without visual symptoms (9.1%, *p* = 0.024). Within the gross total resection group, there was a significant increase in the recurrence rate among patients who presented with visual symptoms compared with those who did not (7.8% at 1 year vs. 3.6%, *p* = 0.009). This association was further reinforced by another study that showed that visual abnormalities at presentation were significantly associated with CP recurrence in children (*p* < 0.001), possibly due to tumor adhesion to the optic nerve or chiasm [62]. On the contrary, other studies performed in pediatric and adult patients showed no statistical significance between visual symptoms, such as chiasmatic syndrome and CP recurrence (*p* = 0.682), or between sixth cranial nerve palsy and recurrence (*p* = 0.09) [47,49], regardless of the higher recurrence rate that was observed in these patients (80% vs. 50%) [30]. These data come in contrast with the study by Duff et al. [32], which demonstrated a significant association between 6th cranial nerve palsy at presentation and CP recurrence (*p* = 0.0337).

Since the hypothalamic–pituitary axis is compromised or compressed by the tumor, symptoms, such as growth failure (75%), delayed puberty (60%), and diabetes insipidus (10–20%), are often reported, while most CP patients suffer from pituitary insufficiency or even panhypopituitarism (75–95%) [127]. When obstructive hydrocephalus is present, symptoms of functional decline manifest, such as psychomotor deficits or school performance decrease, due to frontal lobe compression [127,128]. While hypothalamic disturbance and hormonal-related manifestations have been indicated as risk factors for CP recurrence, very few studies have further investigated the significance of their association. One study in children reported that hypothalamic involvement was significantly associated with tumor recurrence in survival analysis (*p* = 0.01), but no significance was observed when logistic regression was performed (*p* = 0.07) [33]. Other researchers have reported that patients with hypothalamic damage, either by infiltration or compression at presentation, demonstrated more frequent recurrences; however, no further association was made [9,23].

Hormonal-related symptoms are typical manifestations of CP, and their potential role as prognostic factors of tumor recurrence has also been examined, albeit with contradictory results so far. Panhypopituitarism has been documented as a common symptom at presentation and associated with possible recurrence; however, either no statistical significance occurred (Tena-Suck et al.: *p* = 0.191, Rogers et al.: *p* = 1.000) [30,63] or no further analysis was performed [64]. It should be noted, however, that Gautier et al. [24] indicated that the isolated presence of hormonal-related manifestations is associated with a better outcome (OR: 0.38, 95%CI: [0.14–1.03]), while symptoms of raised ICP with a worse one.

### 4.4. Histological Features of the Tumor and Recurrence

Early studies have shown that the adamantinomatous CP tends to be more invasive [27,67] and to form villous elongations into the surrounding brain and particularly in the hypothalamus [39]. Histopathological examination of resected CPs frequently reveals isolated nests of tumor cells extending into, apparently, invading the surrounding gliotic brain tissue [26]. These features make gross-total resection of adamantinomatous CP more difficult. Since partial tumor resection has been linked with higher recurrence rates, it was assumed that these features render adamantinomatous CP a more aggressive and recurrent type [65,66]. However, several recent studies found no significant difference in recurrence rate between the two histological types of CP, and this lack of difference persisted independent of resection status [14,16,26,27,30,32,69,70]. In addition, several recent studies have challenged the claim that finger-like epithelial protrusions are associated with an increased risk of recurrence [26,32].

Since neither the histological type (adamantinomatous vs. papillary) nor the presence of finger-like protrusions seem to increase the tumor’s recurrence risk, other histological features have been investigated as risk factors (Table 1). Among them, the presence of whorl-like arrays has recently been correlated with CP recurrence [30]. The structures are morule-like tumor cell nests that can be identified in histological sections of adamantinomatous CPs. They are thought to be caused by mutations in the β-catenin gene (*CTNNB1*), which are found almost exclusively in adamantinomatous CPs, as shown in a study by Brastianos et al. [129]. Such mutations are important in the Wnt signaling pathway [129,130], which has been shown to act as a promoter of epithelial migration through the regulation of fascin’s gene expression, a protein implicated in filopodia formation [131]. Histologically, clusters of catenin-rich cells have been identified in the tumor–brain border and the tumor’s cyst wall, suggesting a possible role of *CTNNB1* mutations in the aggressive expansion of some adamantinomatous CPs [31,132]. Similarly, a recent study by Guadagno et al. [77] reported that immunohistochemical expression of β-catenin in tumor tissue was strongly associated [*p* = 0.0039] with an increased CP recurrence risk.

Another feature that has been examined relative to the CP’s recurrence risk is the presence of peritumoral gliosis. Reactive gliosis is the proliferation and hypertrophy of glial cells in response to brain tissue damage [133]. More than fifty years ago, Bartlett et al. described that rapidly growing CPs are characterized by a prominent gliotic reaction [72]. Subsequent studies have suggested that the presence of a thick layer of reactive gliosis at the tumor–brain interface may highlight a more aggressive tumor invading the adjacent tissues, with only one showing an increased recurrence risk [27,58,72,134]. On the contrary, a prominent peritumoral gliotic layer has been used as a non-functional dissection plane that facilitates a more extensive dissection of lesions. Indeed, in a study by Weiner et al. [27], 68% of totally removed CPs had a macroscopically visible layer of gliosis around the tumor, compared to 48% of the partially removed tumors. Likewise, several authors have identified the lack of peritumoral gliosis as a major risk factor for multiple subsequent operations [16,23,27,29,65,70].

### 4.5. Molecular Features of the Tumor and Recurrence

Several molecular features of CP have been studied as potential markers of their recurrence (Table 1). It is known that Ki-67 is a protein expressed in mammalian nucleated cells and directly associated with cell proliferation [135]. Extensive literature supports its use as a prognostic marker for tumor staging, assessment of cancer relapse, and prognosis in various tumor types in children and adults [136,137]. Increased Ki-67 expression has been noted in malignant CPs [34]. In addition, studies have examined this molecular index in relation to CP recurrence rate, albeit with conflicting results [16,138]. Nishi et al. [73] and Rodriguez et al. [34], for example, reported a significant correlation between high Ki-67 expression and CP recurrence. Other authors have reported a markedly increased Ki-67 expression in recurrent CP tumors [74,75,76,78], while several others failed to establish such a relationship [14,35,74,79,80,81,82,83]. The wide range of Ki-67 expression in recurrent CPs (0.1–49%) observed in various studies could be partly attributed to the fact that Ki-67 positive nuclei do not show a uniform distribution in each tissue sample [74,81], and on the other, Ki-67 expression may not be constant throughout tumor progression [139]. Prieto et al. [16], in a large systematic analysis of 298 patients from 12 studies, concluded that high Ki-67 expression was among the most reliable tumor markers for predicting an increased risk of recurrence and rapid tumor growth, as long as it was combined with pathological and therapeutic factors, particularly tumor topography and the degree of tumor removal [138].

Protein p53, a regulatory protein of the cell cycle with tumor suppressor properties, has been implicated in the pathogenesis of almost half of all cancers [140]. There are a few studies examining the possible role of altered p53 expression in the aggressivity and relapse of CP with conflicting results. Tena-Suck et al. [30], for example, found that altered immunoreactivity for p53 was significantly (*p* = 0.022) correlated with tumor recurrence or regrowth but without being associated with a specific histopathological subtype. The possible correlation between p53 expression and CP aggressiveness is further supported by the higher expression of p53 in malignant CPs compared to benign CPs [84] and the higher expression of p53 found in recurrent tumor specimens compared to primary CPs [16,36,39]. Nevertheless, there are studies that failed to demonstrate a correlation between p53 immunopositivity and CP histogenesis or recurrence rate, such as the ones by Momota et al. [85] and by Yalçın et al. [82], respectively.

Another molecular marker that, although not predictive of CP recurrence, appears to be of interest is the BRAF gene mutations. A particular mutation, namely, the BRAF p.Val600Glu mutation, has been described as a genetic hallmark of papillary CPs, as it is present in >95% of squamous papillary CPs and, surprisingly, in none of the adamantinomatous CPs [78,129]. This association suggests that activation of the MAPK/ERK pathway leading to suppression of apoptosis is probably the main oncogenic driver of papillary CP [141]. Indeed, BRAF-targeted chemotherapy in patients with papillary CP resulted in a dramatic reduction in tumor volume and cessation of tumor recurrences [142,143,144,145].

The main role of VEGF is the regulation of angiogenesis in both physiologic and pathologic [e.g., tumorigenesis] conditions [146]. Hypoxia-inducible factor 1α (HIF1α) is a transcription factor that regulates the cellular response to hypoxia and seems to be dysregulated in cancer cells [147]. Both of these factors have been examined as potential markers of increased risk of CP recurrence. In a study by Liu et al. [86], for example, CP recurrence was associated with higher VEGF and HIF1α expression, regardless of histopathological subtype, as the relative expression of VEGF and HIF1α in recurrent compared to non-recurrent CPs was 1.07 to 0.32 (*p* = 0.001) and 3.09 to 0.75 (*p* = 0.001), respectively. Similarly, previous studies have shown increased expression of both VEGF and its cellular receptor in recurrent or metastatic CPs, indicating a possible role of VEGF in neo-angiogenesis and tumor regrowth [35,37,87,88]. In another study by Vidal et al. [148], CPs with higher microvessel density were found to regrow more frequently compared to those with lower microvessel density, suggesting that the extent of angiogenesis and, thus, VEGF levels have prognostic value in CP patients. Despite the above data, a study by Xu et al. [89] examined 32 patients with adamantinomatous and 31 patients with papillary CP and found no difference in VEGF expression between the recurrent and non-recurrent CPs (*p* > 0.05).

Another possible marker of increased risk of recurrence is the RARs, which are nuclear receptors involved in epithelial maturation and differentiation. RARs family consists of three different isotypes, namely, alpha (RARα), beta (RARβ), and gamma (RARγ), and the corresponding retinoid X receptor with three subtypes, alpha, beta, and gamma. Two studies have shown a potential correlation between the levels of RARs and the risk of CP recurrence [38,39] since they showed higher expression of RARγ in CPs that recurred within two years of surgical resection. Interestingly, these tumors had lower expression of RARβ. A possible explanation for this discrepancy is the different expression of cathepsins, which are proteinases involved in the potential for local invasion. The different expression of cathepsins, specifically cathepsin D and cathepsin K, seems to contribute to the ability of RARs to influence CP recurrence.

Establishing a link between hormones and CP recurrence remains quite challenging. The most important hormonal mediator in the development and progression of CP seems to be the GH receptor. Indeed, in a study by Hofmann et al. [90], it was observed that CPs with high GH receptor expression had a higher proliferative potential than CPs with low GH receptor expression. Accordingly, the insulin-like growth factor-1 (IGF-1) receptor was shown to be more abundantly expressed in adamantinomatous than in the papillary type of CP, an observation that possibly implicates the IGF-1 receptor in the recurrence of this type of tumor [91]. Other hormones, such as sex steroid hormones and their receptors, as well as leptin and insulin, have not been implicated in increasing the risk of CP recurrence, although results are somewhat conflicting regarding estrogen receptors [90,92].

### 4.6. Therapeutic Approach and Tumor Recurrence

Even though knowledge of the cellular and molecular mechanisms involved in tumor recurrence is limited, several clinical studies have found a significant association between the presence of residual tumor and the risk of CP recurrence in both children and adults [1,13,15,18,19,20,22,23,24,26,27,28,29,30,32,41,44,48,67,69,93,94,95,96,97,99,100] (Table 1). Therefore, a complete tumor resection, which includes resection of the outer tumor capsule adjacent to healthy tissues, is considered to be the best approach in order to minimize the possibility of tumor recurrence [7,149]. In a systematic review by Prieto et al. [16], for example, the mean recurrence rate after a total removal was 23% compared to 63% after a partial removal. The mean time between the first surgery and CP recurrence was also different between the two groups of patients (24 months for total and 45 months for subtotal surgical excision). In recent years, improvements in surgical techniques have increased the frequency with which a complete tumor resection can be achieved without excessive morbidity or mortality [15,41,150]. However, complete CP resection is still only achieved in a percentage of CP cases [41,70,100], and even then, the recurrence risk is high, reaching a 10-year rate of 95% [22]. In addition, an aggressive total resection is usually accompanied by endocrine dysfunction and hypothalamic damage, thus leading to increased morbidity for patients [149]. For these reasons, many authors advocate a less aggressive surgical treatment followed by RT, and in recent years, there has been an increasing tendency toward subtotal resection of complex craniopharyngiomas followed by adjuvant RT to maximize the quality of life while achieving tumor control [1,22]. Studies have shown that tumor control rates after subtotal resection and RT are similar to the ones reported after gross-tumor resection but with lower morbidity [20,100]. Even more controversial is the best approach for a child with recurrent CP. A study by Elliott et al. [15], including 86 children with primary and recurrent CP, showed that gross-total resection was more difficult to achieve in recurrent tumors, especially those with increasing size and after prior RT. Nevertheless, radical resection was still possible in patients with recurrent CPs with morbidity similar to that of primary tumors. Another factor strongly influencing the risk of tumor recurrence, independently of histopathology, both for primary and recurrent tumors, is the ability of the neurosurgeon and their team to achieve gross total resection in candidate patients [16,50,68,71,101,102]. Therefore, an experienced neurosurgical team should be in charge of dealing with these patients.

The use of adjuvant RT is another factor that has been shown in several patient series to significantly reduce the risk of recurrence after subtotal tumor removal [1,19,20,22,23,29,32,40,67,69,93,94,95,96,97,98,103,104,105,106,107,108,109]. In many of these studies that included pediatric patients with CP, the mean recurrence rate of patients treated with RT after subtotal removal was similar and sometimes superior [20] to the one observed in patients after total removal. In a meta-analysis including 442 patients who underwent tumor resection, Yang et al. showed that the 2- and 5-year progression-free survival rates for the gross-total resection group versus the subtotal resection followed by adjuvant RT group were 88 vs. 91%, and 67 vs. 69%, respectively [151]. Similar were the results of another systematic review of a cohort of 531 pediatric CP patients from a total of 109 studies showing similar rates of tumor control with both approaches [21]. These data suggest that subtotal resection followed by adjuvant RT may be equally efficient with gross-total resection without the morbidity associated with aggressive surgical procedures. In addition, contemporary RT techniques permit greater treatment precision and conformity. These approaches decrease but do not eliminate long-term toxicity by limiting the exposure of surrounding normal tissues to ionizing radiation [152]. In addition, there is still no consensus regarding the best irradiation technique, the most appropriate time to administer RT, and at what exact dosage. In addition, some studies have shown that once the pediatric CP recurs, it is exceedingly difficult to treat after prior irradiation since newer RT techniques decrease but do not eliminate long-term toxicity due to the exposure of surrounding normal tissues to ionizing radiation. Therefore, some authors suggest that gross-total resection may need to be the surgical goal at the time of first recurrence, if possible [153]. In total, it is of utmost importance to carefully evaluate each pediatric CP patient to reach the perfect balance between quality of life and the best tumor control approach.

Another factor that has been implicated in the risk of CP recurrence is the absence or presence of tumor calcifications. Elliott et al. [42] showed that minimal residual calcification does not have an impact on the risk of recurrence after gross-total removal in pediatric CPs. Given the potentially harmful effects of RT in the pediatric population, the authors suggest that RT should be withheld in patients after CP gross-total removal and only minimal residual calcification on MR or CT imaging CT, albeit with a close follow-up. In contrast, several authors found that the presence of calcifications in children with CP is an independent risk factor for unsuccessful complete tumor removal and, therefore, for an increased risk of tumor recurrence [28,40,108,110,111]. For example, Fahlbusch et al. [28], in 148 patients with CP who underwent initial (primary) surgery, found that the main reasons for incomplete removal were attachment to and/or infiltration of the hypothalamus, major calcifications, and attachment to vascular structures. Patients with total removal had a recurrence-free survival of 86.9% at 5 years vs. only 48.8% for those with subtotal removal and 41.5% for those with partial removal, implicating the initial presence of calcifications in a higher recurrence rate and lower survival. Similarly, in another cohort of children with CP that were operated on, the absence of calcification on diagnostic neuroimaging (n = 8/30) was significantly associated with improved 5-year progression-free survival (100% vs. 42.9% [SE = 14.7%], *p* = 0.02), even when adjusted for the extent of resection (*p* = 0.03). [40] In a more recent retrospective analysis of the clinical data of 92 children with CP who underwent surgical treatment, the authors found a statistically significant difference (*p* < 0.05) between the degree of tumor calcification and the recurrence rate after the operation and the mortality rate [111]. It, therefore, seems possible that, despite the conventional assumption that residual calcifications do not correspond to viable tumor tissue, their presence is frequently associated with higher rates of partial tumor excision, resulting in residual tumors, which increase the risk of tumor recurrence.

Selection of the right type of surgical approach is also crucial and must ensure complete tumor resection with the least possible damage to the adjacent important neuronal structures [22,41]. To achieve wide exposure of the chiasmatic region, a combination of subfrontal and pterional approaches is usually preferred, performing a frontotemporal method from the nondominant side, extending frontally near the midline [18,100]. The transsphenoidal approach has been used in patients in whom the lesion was exclusively intrasellar or in cases of intrasellar and suprasellar tumor extension with symmetrical and homogeneous intrasellar and suprasellar growth [154,155]. The transcranial approach was selected when the tumor was exclusively suprasellar or in cases of intrasellar and suprasellar extension with asymmetrical and larger suprasellar development [22]. In selected cases, by using the transsphenoidal approach, even tumors with large suprasellar expansion can be managed. The main disadvantage of endoscopic, endonasal, and transsphenoidal surgery, mainly with huge suprasellar expansion, is the increased risk of cerebrospinal fluid leakage [156,157]. Nevertheless, it seems that this approach may give excellent results with minor risks when used in appropriately located craniopharyngiomas and by neurosurgeons with extensive experience in pituitary surgery [158,159]. Recurrence rates associated with the various approaches vary among studies. For example, in a study by Minamida et al. [70], who compared different surgical approaches in 37 consecutive patients with CPs, the recurrence rates were as follows: 20% in patients treated with the basal interhemispheric approach; 25% in those treated with the pterional approach; and 60% in those treated with the transsphenoidal approach.

Safety concerns related to GH treatment and CP recurrence or regrowth come from in vitro studies that have demonstrated the growth of CP cells cultured in the presence of exogenous GH [91], from the identification of GH receptors on CP cells [90], and from the observation that increased GH receptors expression may indicate higher tumor aggressiveness [160]. In addition, there are a few case reports describing a rapid CP enlargement after GH therapy initiation [112,113]. On the contrary, robust data come from carefully conducted case-control studies in pediatric or mixed pediatric–adult populations, most of which have failed to demonstrate any evidence of CP recurrence or regrowth with GH therapy [14,15,43,44,114,115,116,117,118,119,120,121]. Similarly, in adults, a recent well-conducted, retrospective analysis of 89 patients with adult-onset craniopharyngioma with a median follow-up of >7 years demonstrated no increased risk of CP recurrence after surgical excision in those treated with GH [161]. Furthermore, a recent meta-analysis comparing 3436 patients who received GH with 51 who did not [126] demonstrated a protective effect of GH treatment on CP recurrence (overall CP recurrence rate 10.9%, 95%CI: 9.80% vs. 35.2%, 95%CI: 23.1%, for patients with or without GH treatment, respectively; *p* < 0.01). These results, however, may reflect a selection bias in the included studies favoring GH treatment in patients with less aggressive CP. In addition, safety data of GH treatment come from post-marketing surveillance studies sponsored by the pharmaceutical industry, with equally reassuring results [45,122,123,124,125].

## 5. Conclusions

Despite the extensive research conducted over the past four decades on the mechanisms of CP recurrence in children and adolescents, many aspects of this intriguing process remain elusive. Data from large case series and cohort studies are quite often conflicting, mainly due to the heterogeneity of the specific characteristics of each CP in terms of topography, size, adhesiveness, histology, molecular characteristics, long-term behavior, and so forth. Subtotal surgical removal not followed by RT is the predictor of CP recurrence. Further, a younger age, large cystic tumors, tight adherence to surrounding structures, specific clinical findings at diagnosis, presence of histological whorl-like arrays, and some specific molecular features may all be associated with a higher rate of CP recurrence.

Systematic reviews and meta-analyses of each of the individual factors examined, but mostly, well-designed multicenter prospective studies with large numbers of CP patients, will shed further light on the pathomechanisms involved. This will not only assist in identifying prognostic factors for the risk of CP recurrence for each individual patient, thus helping in the best treatment therapeutic strategy already at the time of initial diagnosis. It will also guide the development of new targeted adjunct therapies that, together with tumor resection and local RT, will increase recurrence-free survival rates and improve the quality of life of these patients in the long run.

## Data Availability

Not applicable.
